# Clinical Performance of Duritect‐AD^TM^: a Blood‐based Autoantibody Test for the Detection of Alzheimer's Disease‐Related Pathology

**DOI:** 10.1002/alz70856_100979

**Published:** 2025-12-25

**Authors:** Cassandra DeMarshall, Jeffrey Viviano, Anuradha Krishnan, Funda Suer, Mert Sahin, Robert Nagele

**Affiliations:** ^1^ Durin Life Sciences, Stratford, NJ, USA; ^2^ New Jersey Institute for Successful Aging, Rowan University, Stratford, NJ, USA

## Abstract

**Background:**

In the present study we demonstrate the clinical utility of the Duritect‐AD^TM^ test, a customized panel of blood‐based autoantibody biomarkers capable of detecting AD‐related pathological processes throughout the clinical spectrum of AD, beginning with the earliest stages of the disease, including prodromal AD, as well as more advanced stages of mild‐moderate AD using a proprietary risk score capable of predicting an individual's likelihood of developing AD in the future.

**Method:**

Utilizing Duritect‐AD^TM^'s autoantibody biomarkers, 509 sera samples from ADNI subjects with confirmed presymptomatic, prodromal (MCI), and mild‐moderate AD, as well as healthy, cognitively normal control subjects, were screened to detect the presence of AD‐related pathology. Autoantibody levels were evaluated using a proprietary machine learning algorithm to calculate an individual Alzheimer's Disease Risk Score (ADRS) for each patient sample, indicating whether that individual has a typical, or an increased risk of ongoing AD pathology.

**Result:**

Results demonstrate that this panel of autoantibody biomarkers corresponding to a patient's Alzheimer's Disease Risk Score successfully differentiated ADNI subjects from age‐ and sex‐matched controls with markedly greater than 90% accuracy, as well as high sensitivity and specificity.

**Conclusion:**

Duritect‐AD^TM^ is a CLIA/CAP validated blood‐based autoantibody test for the assessment of the risk of AD‐related pathology in patients aged 55 and older presenting in primary care settings with signs or symptoms of cognitive impairment that could indicate suspected AD. Duritect‐AD^TM^ is an accurate, minimally invasive, and inexpensive test that can be used to aid in the detection of early, AD‐related pathology associated with prodromal (MCI) and later stages of AD.

